# Acute Monoblastic Leukemia with Erythrophagocytosis and Absence of KAT6A Rearrangement

**DOI:** 10.4274/tjh.galenos.2020.2020.0237

**Published:** 2020-11-19

**Authors:** Carlos De Miguel Sánchez, Diego Robles de Castro, Ana Vega González de Viñaspre, Ariane Unamunzaga Zilaurren, Arantza Mendizábal Abad, José María Guinea de Castro

**Affiliations:** 1 Hospital Universitario de Álava - Sede Txagorritxu, Department of Hematology, Vitoria-Gasteiz, Spain

**Keywords:** Erythrophagocytosis, Acute myeloid leukemia, Monoblast, t(8;16)(p11.2;p13.3)/KAT6A-CREBBP

A 44-year-old woman presented with fever and arthralgia. Her past medical history included ulcerative colitis, which was treated with azathioprine and infliximab. On admission, a full blood count revealed a hemoglobin level of 13 g/L, leukocyte count of 1.4x10^9^/L, and platelets of 108x10^9^/L. Peripheral blood smear showed 14% blast cells.

The bone marrow smear was hypocellular and revealed 79% large blast cells with rounded nuclear contours, fine chromatin with one to three nucleoli, and basophilic cytoplasm, which were compatible with monoblasts. Erythrophagocytosis was frequently observed in several blasts (Figures 1A and 1B). Hemophagocytosis of platelets was also observed (Figure 1C). Cytochemical staining showed strong alpha-naphthyl acetate esterase activity (Figure 1D). Flow cytometry analysis showed a large blast population with immature monocytoid phenotype (cyMPO±, HLADR+, CD14-, CD33+, CD34-, CD64+, and CD117-). Cytogenetic analysis displayed a null karyotype. Molecular analysis with nested RT-PCR was done in order to dismiss KAT6A-CREBBP gene rearrangement, which was negative. Bone marrow evaluation after induction chemotherapy showed complete morphological remission and normal karyotype.

Erythrophagocytosis by leukemic blasts is an extremely rare phenomenon and is mostly seen in acute myeloid leukemia, especially associated with monocytic differentiation, t(8;16)(p11.2;p13.3)/KAT6A-CREBBP, t(16;21)(p11;q22), and inv8(p11q13) [1,2,3]. Erythrophagocytosis in the case of monoblastic acute leukemia should prompt exploration for t(8;16)(p11.2;p13.3)/KAT6A-CREBBP [1,4].

## Figures and Tables

**Figure 1:**
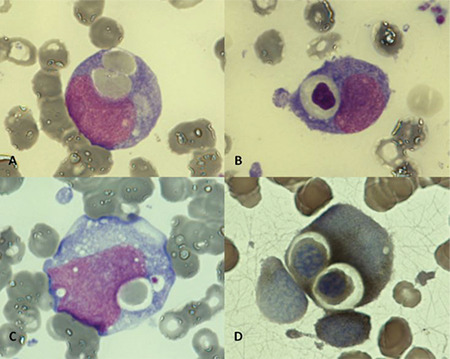
A and B) Erythrophagocytosis was frequently observed in several blasts. C) Hemophagocytosis of platelets. D) Strong alpha-naphthyl acetate esterase activity revealed by cytochemical staining.
